# Development of an improved murine model of necrotizing enterocolitis shows the importance of neutrophils in NEC pathogenesis

**DOI:** 10.1038/s41598-020-65120-y

**Published:** 2020-05-15

**Authors:** Michaela Klinke, Deirdre Vincent, Magdalena Trochimiuk, Birgit Appl, Bastian Tiemann, Konrad Reinshagen, Laia Pagerols Raluy, Michael Boettcher

**Affiliations:** Department of Pediatric Surgery, UKE Medical School, Martinistrasse 52, 20246 Hamburg, Germany

**Keywords:** Experimental models of disease, Infant necrotizing enterocolitis

## Abstract

Various research models to induce necrotizing enterocolitis (NEC) in animals exist, yet significant differences in NEC severity between murine animal models and human patients persist. One possible explanation for the difference in severity may be the variance in neutrophil concentration among newborn humans (50–70%) in comparison to neonatal mice (10–25%). However, neutrophil activity has yet to be evaluated in NEC pathogenesis. Thus, the aim of the study was to evaluate the effects of altered neutrophil concentrations in neonatal mice while simultaneously undergoing a NEC induction. A total of 44 neonatal mice were included in this study and 40 were subjected to an established NEC induction paradigm and 4 were assigned a sham group. Of the 40 mice, 30 received granulocyte-colony stimulating factor (G-CSF) on a daily basis, while 10 were used as controls (receiving inactivated G-CSF). Mice undergoing G-CSF treatment were further divided into two subgroups: (1) wildtype and (2) ELANE-knockout (KO). ELANE - KO mice are incapable of producing neutrophil elastase (NE) and were used to evaluate the role of neutrophils in NEC. For each of the groups, the following metrics were evaluated: survival, NEC severity, tissue damage, neutrophil count and activation, and NETs formation. An improved murine model of NEC was developed using (1) Lipopolysaccharides and Neocate gavage feeding, (2) hypoxia, and (3) G-CSF administration. The results suggest that the addition of G-CSF resulted in significantly elevated NEC manifestation rates with consequent tissue damage and intestinal inflammation, without affecting overall mortality. Animals without functioning NE (ELANE-KO) appeared to have been protected from NEC development. This study supports the importance of neutrophils in NEC pathogenesis. The optimized NEC induction paradigm, using G-CSF administration, resulted in elevated neutrophil counts, resembling those of neonatal humans. Elevation of neutrophil levels significantly improved NEC disease manifestation by modeling human physiology more accurately than current NEC models. Thus, in the future, murine NEC experiments should include the elevation of neutrophil levels to improve the transition of research findings from mice to humans.

## Introduction

Necrotizing enterocolitis (NEC) is a devastating inflammatory disease of the intestine that is predominantly seen in preterm neonates. Although the disease affects up to 12% of premature newborns^1^ and has a mortality rate of around 20%, its manifestation and mortality rate continue to increase^2,3^. Furthermore, NEC is also associated with multiple immediate serious complications, such as death due to sepsis, and long-term complications, including intestinal failure, growth delay, and adverse neurodevelopmental outcomes^4^.

To date, NEC pathogenesis is not entirely understood. It is hypothesized that NEC develops after the onset of enteral feeding, during which bacterial colonization of the intestinal tract occurs. However, this hypothesis is believed to describe a final common pathway of multiple etiologic mechanisms, resulting in NEC. Thus, the pathogenesis is considered to be multifactorial, including immaturity of the intestinal barrier system; ischemic injury of the intestine; and hyperinflammation, due to immaturity of the immune system^5^.

To examine the pathogenesis of NEC, various animal models have been established, with the most commonly used test subjects being mice, due to minimal genetic differences with respect to the immunology between mice and humans^6^. However, even though numerous NEC induction protocols have been developed, most research groups are only able to achieve NEC manifestation rates of roughly 50%^7–9^ and the NEC severity is often milder than the what is observed in humans.

It is hypothesized that the pertinent differences between mice and humans are cause for the low NEC manifestation rates seen in animal models, as well as the often failing translation of animal research models to human patients^10^. In particular, significantly different neutrophil concentration among newborn humans (50–70%) in comparison to neonatal mice (10–25%) could be responsible for the reduced NEC severity observed in mice undergoing NEC induction as compared to human neonatal NEC patients^11^. This is of interest as NEC is considered a hyperinflammation reaction with neutrophil activity being crucial in its pathogenesis^9,12^. In fact, various studies have documented neutrophils’ essential role in NEC pathogenesis, with neutrophil extracellular traps (NETs) being crucial in NEC development^9,13,14^. NETs are web-like DNA structures that neutrophils expel via phagocytosis or during apoptosis (aka NETosis), and are studded with lethal concentrations of antimicrobial proteins and histones, which are able to bind and eliminate microorganisms^15^. Moreover, not only do NETs immobilize pathogens, but they have also been shown to modulate mechanisms of thrombosis during i.e. sepsis, also known as immunothrombosis^15–17^. Even though the formation of NETs is an innate immune mechanism to combat infection, NETs formation can occur during sterile inflammation, autoimmunity, and ischemia reperfusion injury^9,18–21^. Thus, as NEC involves a hyperinflammation reactions, as well as immunothrombosis, an essential role of NETs in NEC development is very likely, especially as neonates diagnosed with severe NEC show intestinal ischemia, thrombocytopenia, disseminated intravascular coagulopathy (DIC), and severe inflammation^22^.

In order to develop a mouse NEC model, which is more in line with human neutrophil levels, granulocyte-colony stimulating factor (G-CSF) has been utilized in previous research. G-CSF stimulates the bone marrow to produce granulocytes and activate neutrophils^23^. Thus, combining the standardized NEC induction paradigm, as employed by the latest NEC research, with an altered mouse neutrophil concentration, using G-CSF, may (1) yield a better understanding of NEC pathogenesis and (2) could more accurately model human physiology.

## Methods

### Study design

The study was approved by the Hamburg State Administration for animal research (63/16). A total of 44 mice were utilized for the experimental model and were held within the animal facility according to environmental parameters established and dictated by the German guide for the care and use of laboratory animals (Tierschutzgesetz).

### Animal procedures

#### NEC Induction paradigm

NEC was induced using a well-established protocol:^24^ Pregnant C57BL/6 J mice (Wildtype and ELANE-knockout) were singly housed within the animal care facility with food and water ad lib. ELANE-Knockout (ELANE-KO) mice (B6.129×1-Elane, Jackson Laboratory) have a mixed C57BL/6 J; C57BL/6 N background and present with systemic neutropenia caused by heterozygous mutations in a gene formerly known as ELANE, which encodes for neutrophil elastase (NE)^25^.

**Figure 1:**
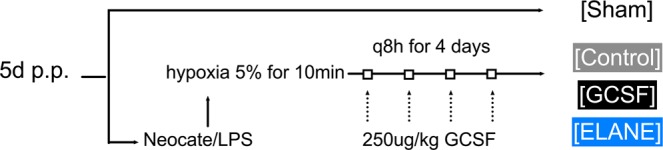
Experimental design and treatment strategy. Mice were subjected to Neocate/LPS gavage feeding followed by hypoxia at 5% for 10 min three times daily for four days. The induction was started on day five p.p. Subjects were treated according to their assigned group with G-CSF (G-CSF and ELANE-KO group) or inactivated G-CSF (control group) via s.c. injections for four days once daily. In order to control for other factors that may influence the pups, excluding the induction paradigm, a sham group was also included.

Mother animals delivered naturally and pups were kept with their mother throughout the entire experimental procedure. On day five postpartum (p.p.), the NEC induction paradigm commenced: Animals were gavage fed a solution of 0.1 ml Neocate (Nutricia) and 4 mg/kg/d lipopolysaccharide (LPS) from E. coli LPS-EB (InvivoGen), using a 1:50 concentration of LPS to Neocate three times daily, followed by 10 minutes (min) of hypoxia at 5% oxygen. The total induction paradigm was maintained for four days and the animals were euthanized on day nine p.p., at the latest. Animals which showed a very high burden under NEC induction were euthanized prior to the predefined endpoint. Furthermore, if animals passed away between the observational intervals (q8h), their tissue samples were not included in the analysis, as autolysis progresses rapidly in affected animals. The induction protocol is shown in Fig. 1.

Animals in the G-CSF and the ELANE-KO group received 250 mg/kg bodyweight G-CSF subcutaneously (s.c.) daily, with the dosage having been deduced from a previous study on mice^26^. Animals assigned to the control group received inactivated G-CSF at the same dose as the G-CSF mice. Inactivation of G-CSF was achieved by heating G-CSF to 100 °C in a hot water bath and maintaining the temperature of 100 °C for one hour^27^. Finally, a sham group of 4 subjects without NEC induction or G-CSF treatment was also included.

During the entire length of the experiment, mother animals received tramadol (Tramal 1 mg/ml, Grünenthal) in their drinking water for pain management of the pups: Tramadol ingested by mother mice is transferred to offspring pups through drinking of breastmilk at 12–14% of its original concentration, which has been shown to ensure appropriate pain management for test subjects^28^.

### Sample collection and storage

#### Blood

Upon euthanasia, blood samples were collected through decapitation of the subjects in test tubes containing ethylenediaminetetraacetic acid and processed immediately: The samples were centrifuged at 2000 relative centrifugal force for 10 minutes (min) at room temperature (RT) in order to separate the blood sample into (1) plasma, (2) a buffy coat, and (3) erythrocytes. All entities were preserved at −80°C until further analysis. Ultimately, leucocyte and neutrophil counts were assessed via FACS within the buffy coat layer.

### Intestinal tissue

After blood collection, animals were dissected using a midline incision. Bowel preparation, removal, and assessment was conducted by two observers blinded to the subject’s test group with aid of a light microscope. The small and large intestine was evaluated for macroscopic markers of NEC: (0) no NEC manifestation, (1) pneumatosis intestinalis, and (2) necrosis and perforation. At this time, the morphologic analysis was performed and captured using a 4 K/12-megapixel camera. Following, all relevant segments of the small intestine were evenly distributed into test tubes containing: (1) phosphate buffered saline (PBS) and (2) formalin solution. Tissue stored in PBS was used directly to visualize extracellular traps.

### Tissue preparation and evaluation

#### Extracellular DNA

Intestinal samples were placed in PBS and stained using SYTOX Orange (50 µM, Life Technologies) to analyze extracellular DNA, including NETs and to a lesser degree mitochondrial DNA (mDNA) and nuclear DNA (nDNA) under a fluorescence microscope. As mDNA and nDNA only make up a small fraction of extracellular DNA (QUOTE), we assumed the visualized extracellular DNA was composed primarily of NETs. Images were captured using an Olympus SC 50 camera and digitized with cellSens Standard (Olympus). Scoring was conducted by two observers as follows:

- None (0) – no signs of NETs
- Mild (1) – small amount of NETs
- Moderate (2) – medium amount of NETs
- Severe (3) – large amount of NETs

### Histological analysis

Upon tissue fixation in formalin solution, intestinal samples were dehydrated overnight and embedded in paraffin. Prepared intestinal tissue was cut into 3 µm thick sections and applied to slides for further analysis. All samples were analyzed in a blinded fashion and captured using an Olympus SC 50 camera. Digitization was completed with the cellSens Standard program (Olympus).

### Hematoxylin and Eosin (H&E)

H&E staining occurred using a machine and with a standardized staining procedure, while semi-quantitative assessment of the intestinal damage was conducted under light microscopy. The severity of intestinal lesions was assessed by two investigators blinded to the study groups. Scoring was performed utilizing the previously validated Caplan score^29^.

- Grade 0: intact villi
- Grade 1: superficial epithelial cell sloughing
- Grade 2: mid-villous necrosis
- Grade 3: complete villous necrosis
- Grade 4: transmural necrosis

### Lymphocyte Antigen 6 Complex Locus G6D (Ly6G) Staining

Ly6G staining occurred using a machine and with a standardized staining procedure, while semi-quantitative assessment of the intestinal damage was conducted under light microscopy. Expression was evaluated in a semi-quantitative fashion by two blinded observers:

- None (0) – no signs of tissue staining
- Mild (1) – small amount of tissue staining
- Moderate (2) – medium amount of tissue staining
- Severe (3) – large amount of tissue staining

### Immunofluorescence staining

3μm-paraffin tissue sections underwent a deparaffinization and rehydration process, followed by immunfluorescence staining for DNA (4’,6-diamidino-2-phenylindole – DAPI) and assessment of neutrophil elastase (NE), myeloperoxidase (MPO), and citrullinated histone 3 (H3cit): MPO and NE are markers of neutrophil activation, and H3cit is considered to be the most specific marker of NETs formation^30^. Antigen retrieval was visualized by incubating murine specimen with Target Retrieval Solution pH6 (Dako, Santa Clara, USA) for 90 min at 60 °C, following a cooling step of 30 min. After rinsing sections 2×3 min with a solution of tri-buffered saline and polysorbate 20 (Tween 20) (TBST), blocking of the probes was performed with Protein Block Solution (BioGenex, Fremont, USA) for 30 min at room temperature (RT). Tissue specimen were further incubated with either isotype- or antigen-specific-antibodies at 4 °C (Abcam, UK): Rabbit anti-mouse NE antibody was used at a dilution of 1:200, whereas mouse anti-mouse MPO- and rabbit anti-mouse H3cit- antibodies were diluted 1:50. 18 hours later, sections were rinsed 3×3 min with TBST and subsequently incubated 1:200 with AlexaFluor647- or FITC-coupled secondary antibodies at RT for 30 min (Abcam, UK). After a 3×5 min rinsing-step with TBST, nuclei were counterstained by incubating probes with DAPI for 5 min at RT. Finally, slides were rinsed 5 min with PBS followed by a 5 min rinse with H_2_O, and mounted with Fluoromount-G (Southern Biotech, Birmingham, USA). Digitized MPO-, NE-, and H3cit-stained immunofluorescence slides were then evaluated in a semi-quantitative fashion by two blinded observers:

- None (0) – no signs of tissue staining
- Mild (1) – small amount of tissue staining
- Moderate (2) – medium amount of tissue staining
- Severe (3) – large amount of tissue staining

### Statistics

All data was analyzed using SPSS Statistics 26 (IBM, NY, USA) and GraphPad Prism 8 (GraphPad, CA, USA). A pre-power study calculation was performed using G*Power 3.1, while the power was deducted from a previous study examining NEC in mice [9]. Differences between groups were calculated using the ANOVA and t-test with Bonferroni correction. Results are presented as mean ± standard deviation (SD). For ordinal data, differences were calculated with the Mann-Whitney test. The level of significance for all tests was set at <0.05.

## Results

### Neutrophil levels

Non ELANE-KO animals subjected to daily G-CSF injections demonstrated a significant increase in white blood cell count in comparison to control animals subjected to inactivated G-CSF treatment (4.85 (1.59) K/ul vs. G-CSF 10.27 K/ul (3.12), p = 0.029). In particular, neutrophil counts, among animals receiving G-CSF, showed a considerable rise (inactivated G-CSF 0.68 (0.20) K/ul vs. G-CSF 3.79 K/ul (0.81), p = 0.003). Thus, neutrophil concentrations within the G-CSF non ELANE-KO group were raised from 14% to 36%, resulting in a better reflection of human neonatal neutrophil levels, which are roughly 50–70%^11^.

### Survival and NEC manifestation

**Figure 2:**
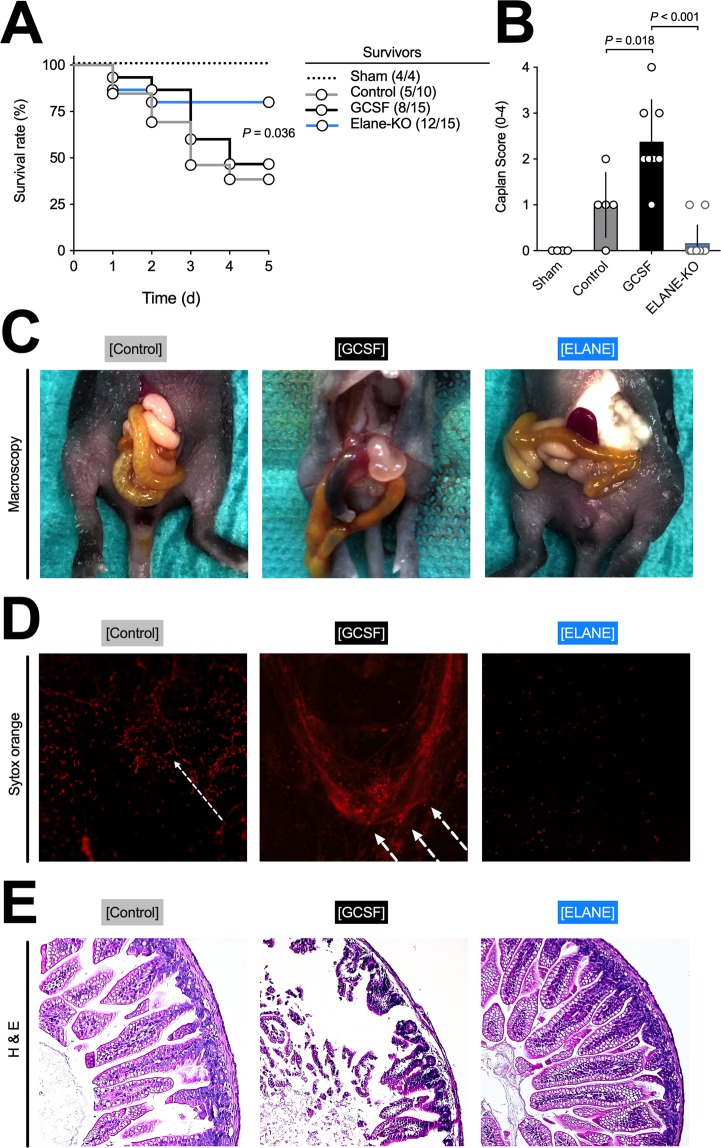
Neutrophilia by G-CSF administration increases NEC manifestation. (**A**) Survival of mice within the NEC groups was not affected by G-CSF administration. However, animals in the ELANE-KO study group were protected from NEC, and showed greater rates of survival. (**B**,**E**) SYTOX Orange staining showed significantly elevated extracellular DNA levels, including NETs, in animals that received G-CSF compared to controls. In animals not receiving G-CSF, SYTOX Orange staining of extracellular DNA was inadmissible. (**C**,**F**) The Caplan score is used to assess intestinal tissue damage in NEC, with our results suggesting that G-CSF administration vastly increased intestinal damage upon NEC induction. (**D**) Macroscopic investigations showed different levels of NEC manifestation. Animals of control group showed light or no signs of NEC similar to animals of ELANE group. Animals receiving G-CSF showed light or advanced signs of NEC. Data shown as Mean±SD. Statistics: Mantel Cox or Mann-Whitney test.

The first endpoint of interest was survival: Controls and animals that received G-CSF demonstrated a similar mortality rate, while animals without functioning NE (ELANE-KO) had significantly higher survival rates. These findings underline the importance of the role of neutrophil activation in NEC pathogenesis (Fig. 2A).

In terms of morphological NEC manifestation (Fig. 2C), all but one animal in the G-CSF group showed light (pneumatosis intestinalis) or advanced (necrosis, perforation, Fig. 2C, middle) signs of NEC (5/8 and 3/8 mice respectively). In the control group, all animals had either light signs (4/5) or no signs (1/5) of NEC (Fig. 2C, left). Thus, G-CSF injections significantly increased NEC manifestation and severity compared to control animals (p = 0.011). In line with these findings, only three out of 12 animals within the ELANE-KO group showed light signs of NEC (Fig. 2C, right).

Similar results were found using the Caplan score (Fig. 2B), assessing for histological NEC manifestation: animals that received G-CSF (Fig. 2E, middle) presented with significantly more tissue damage and higher Caplan scores than controls (p = 0.018, Fig. 2E, left), confirming the increase in NEC manifestation in animals subjected to G-CSF injections. Again, ELANE-KO mice displayed little to no microscopic intestinal injury (Fig. 2E, right).

### Inflammation

**Figure 3:**
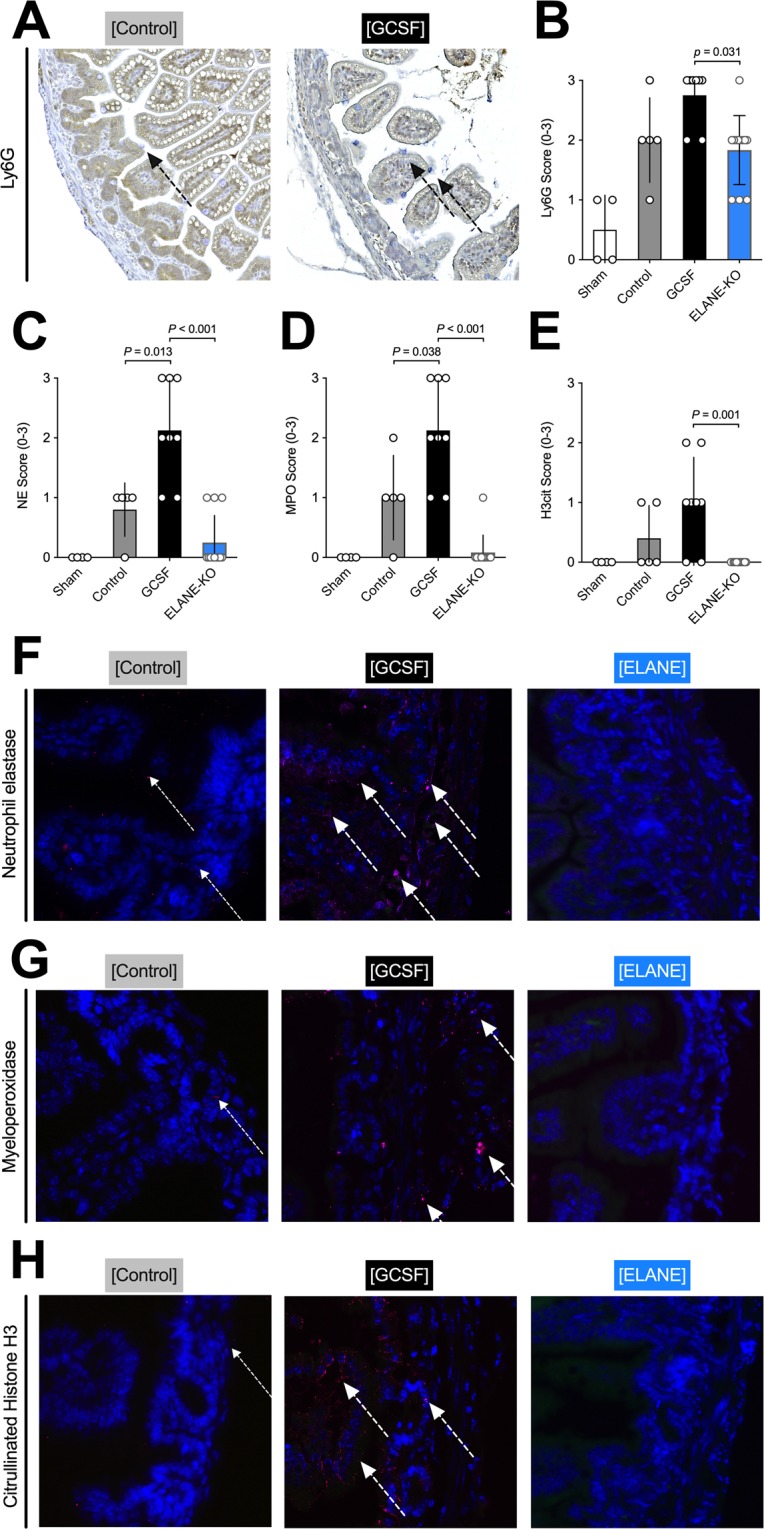
G-CSF administration increases intestinal neutrophil count, activation, and NETs formation. (**A**,**B**) Intestinal neutrophil count did not differ significantly between G-CSF and controls. **(C**,**D**,**F**,**G)** G-CSF treatment resulted in a significant increase in neutrophil activation (NE, MPO) compared to controls. (**E**,**H**) G-CSF administration significantly increasing NET formation compared to controls as indicated by the increased intestinal H3cit levels. In animals without functioning neutrophil elastase (ELANE-KO) neither neutrophil activation nor NETs formation was observed. Data shown as Mean±SD. Statistics: Mann-Whitney test.

In terms of inflammation, animals that received daily G-CSF injections expressed elevated levels of organized extracellular DNA (Fig. 2D, middle) as seen by an increase in staining patterns with SYTOX Orange, as compared to controls (p = 0.049). On the contrary, intestinal samples of animals in the ELANE-KO group hardly showed any extracellular DNA formations (Fig. 2D, right). Coinciding, patterns and concentrations of intestinal neutrophils, as measured by Ly6G staining, were similar in G-CSF and controls, but significantly elevated in G-CSF treated animals, when compared to ELANE-KO mice (Fig. 3A). Moreover, markers of neutrophil activation, namely NE (Fig. 3F) and MPO (Fig. 3D/G), were significantly elevated in animals that received G-CSF compared to controls (p = 0.013 and p = 0.038 respectively). In conclusion, H3cit, a surrogate marker for NETs formation was further elevated in G-CSF treated animals in comparison to controls (Fig. 3E/H). Hence, animals undergoing G-CSF treatment and NEC induction showed changes in line with the pathophysiology observed in neonates with NEC^9^.

## Discussion

As previous NEC animal studies tend to result in low NEC manifestation rates, the aim of this study was to improve current NEC animal models and to evaluate the importance of neutrophils in NEC pathogenesis. Thus, this study aimed to test a potentially improved NEC model with increased NEC manifestation rates through the use of: (1) LPS and Neocate gavage feeding, (2) hypoxia, and (3) G-CSF administration. Adding G-CSF injections to an already established NEC induction protocol led to increased NEC presentation among subjects, including typical signs of intestinal injury, neutrophil activation, and NETs formation. However, despite an amplification of NEC manifestation and presentation under G-CSF application, mortality rates were not affected and were in line with the previously validated NEC induction paradigm. Adding to our improved NEC animal induction paradigm, is the fact that maternal separation and hypothermia, two measures to intensify NEC manifestation in murine pups, were not necessary. Hence, this updated NEC model is a simple alteration to the established models and it effectively reduces procedural burdens on subject animals^7–9,31^.

Our results also suggest that adjusting mice’s (1) neutrophil levels and (2) neutrophil activation during NEC manifestation to levels exhibited by neonates is a key component in establishing a more physiological accurate neonatal NEC mouse model. In the past, various strategies have been utilized to develop animal models that mimic human NEC. One of the most successful NEC induction models, developed by Jilling *et al*., involves exposing preterm pups, delivered by cesarean section, to (1) formula feeding, (2) hypoxia, and (3) hypothermia. Still, even this model, which involves a complicated implementation, as well as questionable animal testing procedures, only resulted in a NEC manifestation rate of 66%^7^. Moreover, Jilling *et al*.’s hypoxia-hypothermia-formula-feeding (HHF) NEC model was subsequently replicated in preterm and term mice by other researchers, and resulted in even lower NEC manifestation rates (around 50%)^32^.

With uncertainties surrounding the reproducibility and efficacy of the HHF model, as well as concerns regarding the lack of translation to human patients, several new models have been developed that attempted to utilize inflammatory pathways rather than hypoxia to initiate NEC injuries. One example is the administration of trinitrobenzenesulfonic acid to ten day old mice, which acts as a hapten, binding to host proteins and generating an immune response that results in a mucosal injury, similar to that seen in NEC patients^33^. A second model, which has also produced intestinal damage analogous to human NEC patients, utilizes a disruption of Paneth cells, followed by enteral gavage feeding of bacteria of 14 day old mice^34^. Both of these models offer additional insights into mechanistic pathways of how the immature intestinal tract can be injured, and strongly support the new theory that NEC may be the final common pathway of multiple initiating events.

The model we created not only aimed to optimize pre-existing NEC models and NEC manifestation rates, but also focused on inflammation of the intestine, rather than hypoxia and reperfusion injury, by altering neutrophil concentrations and, thus, the inflammation cascade. Alteration of neutrophil concentrations was obtained through G-CSF application, which is a cytokine shown to directly target neutrophil biology by (1) increasing the number of circulating neutrophils, (2) augmenting neutrophils’ responsiveness to endotoxin and cytokines, and (3) priming neutrophil effector function^35^. In fact, recombinant human G-CSF, an FDA approved drug, is frequently used to prevent and treat neutropenia in oncological patients^36^. However, in line with neutrophils’ role as first line responders to pathogens, administration of G-CSF does not seem to improve outcomes in adult patients with sepsis. This has been demonstrated from multiple randomized controlled trials^37^. Opposing these findings is the observation that neonates have differently, as studies have found that G-CSF administration improved survival in neonatal patients diagnosed with sepsis in combination with neutropenia^38^.

With respect to NEC development and neutrophils, our study suggests that neutrophils play an important role in the pathogenesis, however, their function remains unclear. A 2011 study conducted by Emami *et al*. describes a NEC induction model involving oral feeding of Cronobacter sakazakii. Their results demonstrated a depletion of both neutrophils and macrophages in the intestinal lamina propria, which caused an increased production of pro-inflammatory cytokines and enterocyte apoptosis, thereby exacerbating the disease^39^. This suggests that both macrophages and neutrophils play an important role in early infection and their absence intensifies the inflammatory response observed in NEC. Thus, in line with the above findings, Kocherlakota *et al*. administered G-CSF to neonates with diagnostically verified NEC. However, instead of an improvement of symptoms, the outcomes were more adverse and the mortality rate elevated^40^. These outcomes are congruent with our results, in that animals that received G-CSF had a much higher NEC manifestation rate with increased intestinal damage, neutrophil activation, and NETs formation. What is more, animals without functioning neutrophil elastase (ELANE-KO) were almost completely protected from NEC, which may indicate that NEC is indeed the result of hyperinflammation of the immature intestine^41^.

Contradicting our findings, however, is the fact that diagnosed neutropenia in neonates with NEC is associated with adverse outcomes^42^. A possible explanation for these conflicting findings could be that neutropenia is not the precursor of NEC, but rather a consequence, due to depletion of the circulating neutrophil pool after emigration into the intestines and peritoneum^42^. Making the assessment of neutrophils’ role in NEC even more complicated is that prematurely born neonates express a broader range of neutrophil levels in comparison to term born neonates. In a study conducted by Schmutz et al. reference ranges for absolute neutrophil counts (ANC), using a large data set of 30,354 complete blood counts from neonates born between 23–42 weeks’ gestation, were compiled. Their results showed that in the interval between 72 and 240 hours after birth, the ANC ranged between 2700–13,000/μL (5^th^–95^th^ percentile) for infants >36 weeks’ gestation, and 1300–15,300/μL at <28 weeks’ gestation^43^. In light of these findings, an assessment of neutrophil counts prior to the onset of NEC in neonates would help distinguish their role in NEC development.

Even though our findings are promising, the current study has some limitations that merit discussion. First, the genetic background of the ELANE-KO mice is slightly different from all other animals: A 32 single nucleotide polymorphism panel analysis, with 27 markers covering all 19 chromosomes and the X chromosome, as well as 5 markers that distinguish between the C57BL/6 J and C57BL/6 N sub-strains, was performed on the rederived living colony at the Jackson Laboratory repository. While all 27 markers throughout the genome suggested a C57BL/6 genetic background, at least 2 of 5 markers that determine C57BL/6 J from C57BL/6 N were found to be segregating. This may have influenced our results and impacted NEC manifestation. Second, the administration of tramadol may have influenced our results, as tramadol not only has antinociceptive, but also anti-inflammatory effects^44^. However, within our study the medication was used in all groups and should have affected all animals similarly, provided that they were able to drink from their mothers. In future studies involving inflammatory pathways, another method of pain management should be considered.

In conclusion, it is unknown whether lower or higher neutrophil levels increase the risk of NEC onset and development to date. An answer to this question would significantly change NEC diagnosis and management, as well as consequent survival and disability of NEC patients. Shedding light on NEC pathogenesis are our results suggesting that increased activation and total number of ANC is associated with a higher risk of NEC development, which is also in line with Kocherlakota *et al*.’s findings, that adverse outcomes are increased in neonates with NEC after G-CSF treatment^40^. However, as there is a vast range of research findings with regards to neutrophils and NEC, further studies are necessary to validate our findings and fully understand the role of neutrophils in NEC.

With respect to our modified animal NEC model, our results suggest that in mice undergoing a NEC induction paradigm, an elevation of neutrophil levels and their consequent activation to levels observed in neonatal humans, improved disease manifestation. Even more, histological changes, observed in G-CSF treated NEC mice, were near identical to those seen in human NEC patients, making G-CSF administration a good addition to already existing NEC models. Future studies should be conducted in order to validate and improve this murine NEC model, by i.e. making use of permanent overactivation of G-CSF through hydrodynamic gene delivery, and therefore replacing the need for daily subcutaneous G-CSF administration.
